# Characterizing the Healthcare Utilization and Costs of Hereditary Hemorrhagic Telangiectasia

**DOI:** 10.1002/ajh.27756

**Published:** 2025-07-02

**Authors:** Hanny Al‐Samkari, Tracy J. Mayne, Misty Troutt, Hemant Patle, Marianne Clancy, Eric Duhaime

**Affiliations:** ^1^ Division of Hematology Oncology Massachusetts General Hospital Boston Massachusetts USA; ^2^ Slipstream IT Blue Bell Pennsylvania USA; ^3^ Cure HHT Monkton Maryland USA; ^4^ Diagonal Therapeutics Watertown Massachusetts USA

**Keywords:** anemia, bleeding, cost, epistaxis, gastrointestinal bleeding, hereditary hemorrhagic telangiectasia, iron deficiency, Osler‐Weber‐Rendu

## Abstract

Hereditary hemorrhagic telangiectasia (HHT) is the second‐most common inherited bleeding disorder worldwide, afflicting one in 4000–5000 people, and is the most morbid inherited bleeding disorder of women. HHT causes recurrent severe epistaxis, chronic gastrointestinal bleeding, heavy menstrual bleeding, and arteriovenous malformations in the lung, liver, and brain that cause serious bleeding and nonbleeding complications. There are no approved treatments worldwide, and the direct medical costs of HHT have not been well‐characterized. We utilized the Komodo Healthcare Map claims database to create a large sample of US patients diagnosed with HHT, including a subgroup with anemia and a subgroup receiving hematologic support (iron infusions and/or red cell transfusions). We quantified mean per patient per year (PPPY) and total population inpatient, outpatient, and pharmacy costs in 2022 and 2023. The mean PPPY costs for all HHT patients (*n* = 24 407; *n* = 23 524), those with anemia (*n* = 13 856; *n* = 13 192) and those receiving hematologic support (*n* = 6191; *n* = 5818) were approximately $19 000, $27 000, and $40 000, respectively, across years, representing > $450 000 000 in annual healthcare costs in the sample. The leading cost drivers were related to treatment for bleeding and its consequences. While accounting for nearly 60% of HHT patients, those with anemia accounted for ~80% of direct medical costs. Across the majority of leading inpatient, outpatient, and pharmacy cost drivers, patients with anemia and anemia treatment accounted for 75%–100% of cost. The PPPY costs of HHT are comparable to, or exceed, those of other rare, resource‐intensive serious diseases, including sickle cell disease, muscular dystrophy, and cystic fibrosis.

## Graphical abstract

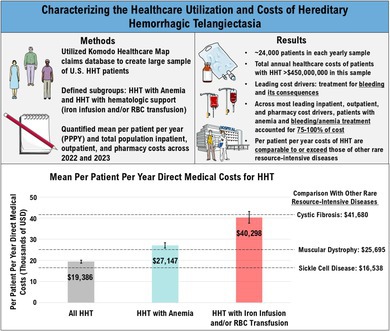

## 1 Introduction

Hereditary hemorrhagic telangiectasia (HHT) is the second‐most‐common inherited bleeding disorder worldwide, afflicting 1 in 4000 to 5000 people, and is the most morbid inherited bleeding disorder of women [1, 2]. HHT afflicts approximately 80,000 people in the United States and 1.4 million people worldwide. HHT results from loss of function mutations affecting transforming growth factor‐β (TGF‐β)/BMP signaling, resulting in chronic angiogenic dysregulation [3, 4]. This causes formation of fragile, bleeding mucocutaneous telangiectasias and arteriovenous malformations (AVMs) in the liver (~70%); lung (~50%); GI tract (~75%); and brain (~20%) [5]. The cardinal manifestation of HHT is recurrent, often severe epistaxis, occurring in > 95% of patients with HHT [6]. Bleeding from visceral and central nervous system (CNS) AVMs can result in hemorrhagic stroke in the brain or spine, pulmonary hemorrhage, and chronic gastrointestinal bleeding, the latter of which occurs in one‐third of all patients with HHT. Visceral and CNS AVMs may also result in numerous nonbleeding complications including stroke, brain abscess, epilepsy, hepatic and pulmonary disease, and high output heart failure [3, 4, 5]. Chronic bleeding in HHT causes iron deficiency anemia in over half of patients, often requiring treatment with iron infusions and/or red cell transfusions [7, 8]. As a result of these varied and morbid bleeding and nonbleeding sequelae, patients with HHT have reduced survival compared with the general population [9]. Despite the severity of HHT, there are no US Food and Drug Administration (FDA) or European Medicines Agency (EMA) approved treatments for HHT, resulting in off‐label use of antiangiogenic drugs such as bevacizumab and pomalidomide to attempt to control disease manifestations [10, 11, 12, 13, 14, 15].

Despite these serious comorbidities, the healthcare resource utilization and costs of treating the consequences of HHT have not been well characterized. A study examining 3977 newly diagnosed patients with HHT between 2007 and 2017 using data from the OptumLabs Data Warehouse reported median 1st‐year costs of $4333 for a commercial insurer and $7333 for a Medicare Advantage plan [16]. Mean, variance, and range were not reported. Compared to matched controls, patients with HHT had more frequent outpatient visits, hospitalizations, endoscopies, iron infusions, and red cell transfusions, though associated costs were not reported. A study of prevalent patients with HHT with data from the State of Florida's Medicare/Medicaid program (2007–2012) and the IBM MarketScan Research Database (2006–2020) reported mean costs of $5590 and $14,265 per patient per year (PPPY), respectively [17]. There was no further description of specific cost contribution. With a recent prevalence estimate of approximately one in 4000 persons [18], the mean annual direct medical costs of HHT could exceed $1 billion in the United States alone.

The objective of this study was to better characterize the direct medical costs associated with treating patients with HHT and defining the primary drivers for inpatient, outpatient, and pharmacy healthcare resource utilization and cost.

## 2 Methods

### 2.1 Data Source

All analyses utilized the Komodo Healthcare Map claims database. The database includes more than 330 million patients in the United States. Claims data are submitted from multiple insurers including commercial, Medicare and Medicaid plans. Komodo uses Datavant deterministic tokenization to link patients across participating insurers. Identifying information is transformed into a unique, encrypted, and nonidentifiable patient token, such that data remain deidentified and HIPPA compliant at all times.

The database includes patient demographics (age, sex, and three‐digit zip code) and insurance enrollment and disenrollment dates. Inpatient claims include care setting (hospital, skilled nursing, inpatient psychiatric and hospice), admission and all secondary International Classification of Diseases, 10th Revision (ICD 10) diagnoses and procedures (ICD 10 Procedure Coding System [PCS]; Healthcare Common Procedure Coding System [HCPCS]; and Current Procedural Terminology [CPT]). Outpatient claims include provider, provider specialty (self‐reported), service setting (physician office, emergency room without admission, ambulatory care), all diagnosis and procedure codes. Pharmacy claims include medication (National Drug Code [NDC]); dose; quantity dispensed; prescription fill date, and actual reimbursement.

The study utilized closed claims, that is, deduplicated claims accepted by and paid by health insurance companies. While the data feed is daily, complete data are generally available at 3–6 months to allow for adjudication. Cost data include reimbursed amounts where available and otherwise incorporate Medicare Allowable Payments.

### 2.2 Sample Selection

The analyses utilized data from patients in the database between January 1, 2018 through December 31, 2023. Patients were required to have an ICD 10 diagnostic code of I78.0: HHT. To eliminate patients with a provisional or “rule out” diagnosis, we required patients to have either a hospital admission with ICD 10 I78.0, or two or more outpatient visits on separate days during the observation period. The ICD 10 PCS, CPT, and HCPCS codes used to define anemia and hematologic support (intravenous iron and red cell transfusion) are shown in Table S1.

To evaluate subpopulations of patients with HHT with anemia and receiving hematologic support, the data dictionary from Komodo's MapLab was reviewed for all codes related to anemia, blood transfusion and iron infusion in the ICD10, CPT, HCPCS, PCS and billing revenue categories for the dataset. These were then applied to the variables of “primary diagnosis code/array,” “admission diagnosis code,” “diagnosis/secondary diagnosis code,” “icd pcs codes,” “cpt hcpcs codes,” “procedure code,” and “revenue codes” within the HHT population to create the subpopulations for analysis.

### 2.3 Analysis

We analyzed costs separately for calendar years 2022 and 2023. We calculated all costs for prevalent patients diagnosed with HHT during the observation period, regardless of whether those costs were specifically associated with an ICD 10 I78.0 diagnosis. Inpatient costs were derived from Diagnosis Related Group (DRG) Medicare Allowable Costs. Outpatient costs were derived from PCS, CPT, and HCPCS. Pharmacy costs utilized NDCs. Costs were calculated as mean ± standard deviation and median PPPY, as well as total cost for the full sample in each year. Costs were reported for all HHT patients, the subset with anemia, and the subset receiving hematologic support, separately by calendar year. Codes used to define anemia and hematologic support are shown in Table S1. We examined the leading costs within each utilization category: inpatient costs by admission ICD 10 code and by DRG; outpatient costs by procedure code; and pharmacy costs by indication category and generic drug name. Note that outpatient procedure codes were consolidated, as differentiation of iron infusion by specific iron product or bevacizumab by branded or biosimilar were not deemed relevant.

## 3 Results

### 3.1 Sample Selection and Characteristics

From January 1, 2018 and December 31, 2023 there were 270 126 unique patients with at least one HHT diagnosis. Of these, 39.2% had two or more outpatient diagnoses on separate days. All patients with an inpatient diagnosis also had two or more outpatient diagnoses, so this criterion did not affect the sample. The number of patients meeting inclusion criteria in 2022 and 2023 were 24 407 and 23 524, respectively.

The demographic characteristics of the sample were consistent across years (Table 1). The sample was predominantly female (61%); the majority of patients were over the age of 50 (approximately one‐quarter were 50–64 years old, 41%–42% were ≥ 65). Just under half of patients had commercial insurance (48%), followed by Medicare (40%), and Medicaid (12%). Patients with anemia did not differ from the overall population on the basis of gender but were more likely to be older and more likely to have Medicare as their primary insurance (Table 1). The same age and Medicare pattern was observed in patients receiving hematologic support (iron infusion and/or red cell transfusion).

**TABLE 1:** Demographic characteristics of patients with HHT and subgroups with anemia and hematologic support in 2022 and 2023.

	2022			2023		
	All HHT patients	Anemia	Hematologic support	All HHT patients	Anemia	Hematologic support
	*N* = 24 407 (100%)	*N* = 13 856 (57%)	*N* = 6102 (25%)	*N* = 23 524 (100%)	*N* = 13 192 (56%)	*N* = 5726 (24%)
Sex (% female)	61	61	59	61	61	60
Age (%)						
< 18	6	2	1	7	2	1
18–29	7	4	3	7	4	3
30–49	18	15	15	18	16	15
35–64	25	27	30	26	28	31
≥ 65	42	51	51	41	50	49
Health insurance (%)						
Medicare	40	50	52	40	50	51
Commercial	48	39	36	48	40	37
Medicaid	12	11	12	12	10	12

*Note*: Hematologic support is defined as intravenous iron infusion and/or red blood cell transfusion.

**FIGURE 1:**
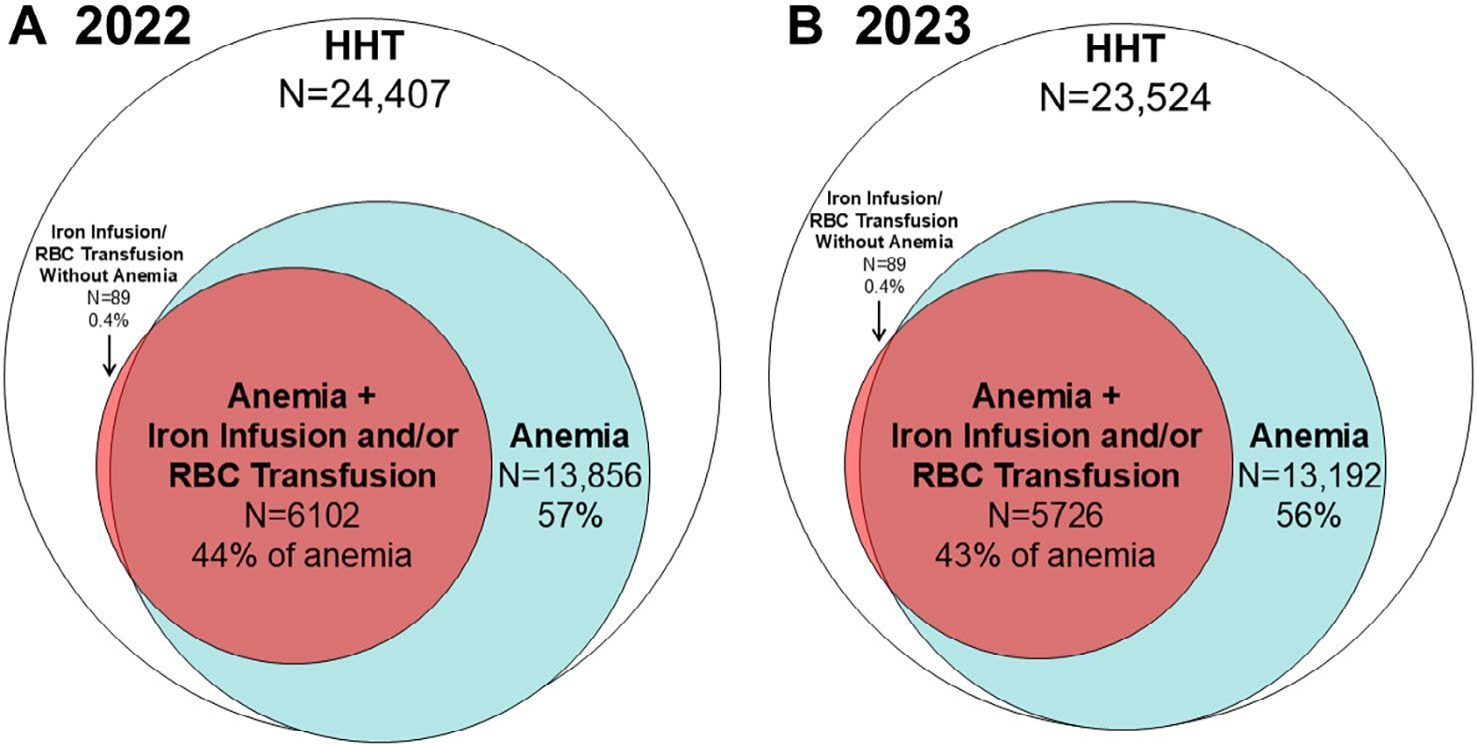
Proportion of patients with HHT with comorbid anemia and hematologic support with iron infusion and/or red blood cell (RBC) transfusion in 2022 (A) and 2023 (B). [Color figure can be viewed at wileyonlinelibrary.com]

Figure 1 shows the number and proportion of patients with HHT with comorbid anemia and those that received hematologic support. The percentage of HHT patients with anemia was 57% in 2022 and 56% in 2023. Nearly half of the patients with anemia had received either iron infusion or RBC transfusion (44% and 43%, respectively), with 11 963 iron infusions and 5113 RBC transfusions in 2022; 13 078 iron infusions and 4944 RBC transfusions in 2023.

### 3.2 Overall Cost

The overall direct medical cost of HHT patients and the subgroups of those with anemia and those receiving hematologic support are shown in Table 2. The overall costs for the sample population were $474 million in 2022 and $456 million in 2023. Across years, the majority of the costs were outpatient (60% and 59%), followed by inpatient (23% and 23%) and pharmacy (17% and 18%).

**TABLE 2:** Direct medical costs of patients with HHT and subgroups with anemia and hematologic support in 2022 and 2023: outpatient, inpatient, pharmacy, and total.

Cost	2022			2023		
	All HHT	Anemia	Hematologic support	All HHT	Anemia	Hematologic support
	*n* = 24 407 (100%)	*n* = 13 856 (57%)	*n* = 6102 (25%)	*n* = 23 524 (100%)	*n* = 13 192 (56%)	*n* = 5726 (24%)
Outpatient	$284 584 440	$220 238 928	$141 526 660	$268 327 275	$199 820 539	$118 625 022
% cost	100	77	50	100	75	44
Mean PPPY	$11 660	$15 894	$23 174	$11 407	$15 147	$20 717
SD	$97 309	$128 220	$190 400	$28 263	$33 135	$41 869
Median PPPY	$3375	$5308	$8621	$3598	$5718	$9211
Inpatient	$107 967 258	$94 537 292	$69 574 039	$104 322 120	$89 907 530	$64 010 747
% cost	100	88	64	100	86	61
Mean PPPY	$4424	$6822	$11 393	$4435	$6815	$11 179
SD	$23 898	$30 448	$40 306	$67 009	$32 486	$43 652
Median PPPY	$0	$0	$0	$0	$0	$0
Pharmacy	$80 608 531	$61 402 213	$34 998 286	$83 672 663	$62 416 400	$33 960 491
% cost	100	76	43	100	75	41
Mean PPPY	$3303	$4431	$5731	$3557	$4731	$5931
SD	$36 345	$46 972	$67 838	$32 324	$41 791	$59 837
Median PPPY	$223	$330	$377	$283	$429	$485
Total	$473 160 229	$376 178 433	$246 098 985	$456 322 058	$352 144 469	$216 596 260
% cost	100	80	52	100	77	47
Mean PPPY	$19 386	$27 147	$40 298	$19 398	$26 694	$37 827
SD	$113 132	$148 197	$217 087	$54 719	$68 328	$91 726
Median PPPY	$4969	$8158	$13 787	$5433	$8850	$14 543

*Note*: Hematologic support is defined as intravenous iron infusion and/or red blood cell transfusion.

Abbreviation: PPPY, per patient per year.

While patients with anemia were 57% and 56% of the population in each year, they accounted for 80% and 77% of the overall costs. Patients with anemia accounted for approximately three‐quarters of outpatient and pharmacy costs across years, and 88% and 86% of inpatient costs in 2022 and 2023. Patients receiving hematologic support were 25% and 24% of the population in 2022 and 2023 but accounted for 52% and 47% of the overall costs. The proportion of outpatient (50% and 44%) and pharmacy (43% and 41%) costs were consistent, while proportion of inpatient costs were higher (64% and 61%) in 2022 and 2023, respectively.

### 3.3 Leading Cost Drivers

#### 3.3.1 Outpatient Costs

The leading outpatient costs by year are shown in Table 3. The costs are generally consistent across years, with the exception of recombinant factor VIIa injection. The majority (94%) of this $21 million cost in 2022 was generated by a single patient with uncontrolled gastrointestinal bleeding (and no bleeding disorders other than HHT). Claims indicate multiple interventions were attempted to control the bleeding and that the patient expired in hospital. Recombinant factor VIIa costs in 2023 were negligible. Overall, the outpatient costs are consistent with HHT and associated comorbidities related to bleeding and anemia, including bevacizumab injection, iron infusions, and embolization procedures to control bleeding.

**TABLE 3:** Direct outpatient medical costs in 2022 and 2023 in patients with HHT and subgroups with anemia and IV iron and/or red blood cell transfusion.

Outpatient procedure	2022					2023				
	All HHT	Anemia		Hematologic support		All HHT	Anemia		Hematologic support	
	*n* = 24 407 (100%)	*n* = 13 856 (57%)	%	*n* = 6102 (25%)	%	*n* = 23 524 (100%)	*N* = 13 192 (56%)	%	*n* = 5726 (24%)	%
Factor VIIa injection	$21 213 969	$21 213 969	100	$19 973 349	94	$7360	$7360	100	$1601	22
Outpatient visit	$17 922 384	$12 434 858	69	$12 485 836	70	$17 028 541	$11 614 214	68	$11 668 951	69
Bevacizumab	$10 483 247	$10 385 250	99	$10 385 250	99	$11 060 277	$10 938 163	99	$10 941 154	99
IV iron	$6 173 070	$6 167 089	100	$6 168 898	100	$5 492 420	$5 477 918	100	$5 491 149	100
Embolization procedures to control bleeding	$6 196 608	$3 133 525	51	$3 175 671	51	$6 634 512	$3 342 244	50	$3 363 096	51
Emergency room visit	$5 166 564	$4 004 930	78	$4 016 201	78	$5 032 420	$3 801 692	76	$3 822 343	76
Subsequent hospital observation care	$3 984 304	$3 731 079	94	$3 747 932	94	$3 699 484	$3 459 630	94	$3 470 948	94
Infusions	$2 605 185	$2 550 984	98	$2 549 477	98	$2 613 993	$2 549 477	98	$2 556 408	98
Pembrolizumab injection	$2 059 085	$1 559 737	76	$1 666 014	81	$1 762 577	$1 237 928	70	$1 259 277	71
Physical therapy	$1 898 062	$1 141 533	60	$1 146 491	60	$1 884 313	$1 156 903	61	$1 161 118	62

*Note*: Hematologic support is defined as intravenous iron infusion and/or red blood cell transfusion.

While accounting for 56% and 57% of the population in 2022 and 2023, patients with anemia accounted for 70%–100% of the costs in seven of the nine leading outpatient cost drivers. Nearly all the incremental costs were in patients who received hematologic support. The cost of pembrolizumab, indicated for the treatment of malignancy, is unlikely to be directly related to HHT.

#### 3.3.2 Inpatient Costs

In 2022, 2792 patients (11%) had 5697 hospital admissions. In 2023, 2687 patients (11%) had 5147 admissions. Among patients with anemia, 2306 and 2194 were hospitalized (17%); and among those receiving hematologic support 1517 and 1403 were hospitalized (25%). Table 4 shows inpatient costs categorized by leading admission diagnosis. Across years, leading cost drivers were associated with similar diagnoses: Sepsis; HHT; alcoholic cirrhosis of the liver with ascites; angiodysplasia of the stomach and duodenum with bleeding; brain AVMs, pulmonary AVMs; and posthemorrhagic anemia.

**TABLE 4:** Direct inpatient medical costs by admission diagnosis in 2022 and 2023 in patients with HHT and subgroups with anemia and hematologic support.

Admission diagnosis	2022					2023				
	All HHT	Anemia		Hematologic support		All HHT	Anemia		Hematologic support	
	*n* = 24 407 (100%)	*n* = 13 856 (57%)	%	*n* = 6102 (25%)	%	*n* = 23 524 (100%)	*N* = 13 192 (56%)	%	*n* = 5726 (24%)	%
Sepsis, unspecified organism	$4 611 866	$4 214 297	91	$2 759 510	60	$5 144 481	$4 484 917	87	$3 139 094	61
HHT	$4 092 703	$3 947 259	96	$3 585 090	88	$4 084 084	$3 670 094	90	$3 285 292	80
Alcoholic cirrhosis of liver with ascites	$3 628 203	$3 571 659	98	$3 262 596	90	$2 482 512	$2 470 364	100	$1 890 054	76
COVID 19	$2 589 357	$2 152 616	83	$1 250 019	48	$1 005 270	$821 966	82	$821 966	82
Angiodysplasia, stomach, and duodenum, bleeding	$2 312 237	$2 312 238	100	$2 240 660	97	$2 806 922	$2 806 922	100	$2 398 044	85
Arteriovenous malformation of cerebral vessels	$2 293 155	$1 301 449	57	$1 301 449	57	$1 174 369	$457 405	39	$457 405	39
Acute posthemorrhagic anemia	$1 895 187	$1 895 187	100	$1 836 164	97	$1 645 226	$1 645 226	100	$1 604 291	98
Congenital pulmonary arteriovenous malformation	$1 336 182	$897 653	67	$970 780	73	$1 114 008	$513 498	46	$513 498	46
Hypertensive heart and CKD with HF	$1 308 302	$1 246 304	95	$844 344	65	$2 275 774	$2 189 816	96	$837 442	37
Angiodysplasia of colon, hemorrhage	$1 267 681	$1 267 682	100	$1 227 741	97	$650 832	$650 832	100	$634 762	98
HTN heart disease with HF	$1 231 815	$1 124 799	91	$689 693	56	$1 137 922	$1 028 172	90	$754 149	66
Sepsis due to *Escherichia coli*	$1 002 160	$965 759	96	$894 940	89	$524 797	$524 797	100	$524 797	100
Paroxysmal atrial fibrillation	$753 977	$738 381	98	$738 381	98	$1 113 794	$1 025 444	92	$1 025 444	92
Nonrheumatic mitral (valve) insufficiency	$361 280	$234 886	65	$234 886	65	$1 046 991	$1 046 991	100	$669 538	64
Hepatic encephalopathy	$262 878	$262 878	100	$262 878	100	$1 153 611	$1 153 611	100	$981 013	85
Alcoholic cirrhosis of liver without ascites	$200 408	$200 408	100	$200 408	100	$1 743 550	$1 743 550	100	$1 699 402	97

*Note*: Hematologic support is defined as intravenous iron infusion and/or red blood cell transfusion.

Table 5 shows inpatient costs categorized by DRG, which represents the most expensive diagnosis or procedure that occurred during the hospital stay. Inpatient costs were more consistent across years when categorized by DRG than by admission diagnosis. Cost drivers were led by severe septicemia, liver transplant, cirrhosis and alcoholic hepatitis, and gastrointestinal hemorrhage, all with major complications.

**TABLE 5:** Direct inpatient medical costs by diagnosis related group in 2022 and 2023 in patients with HHT and subgroups with anemia and hematologic support.

Diagnosis‐related group	2022					2023				
	All HHT	Anemia		Hematologic support		All HHT	Anemia		Hematologic support	
	*n* = 24 407 (100%)	*n* = 13 856 (57%)	%	*n* = 6102 (25%)	%	*n* = 23 524 (100%)	*N* = 13 192 (56%)	%	*n* = 5726 (24%)	%
Septicemia/severe sepsis, no mv > 96 h, major complications (mcc)	$4 269 125	$3 940 638	92	$2 935 056	69	$4 995 581	$4 524 451	91	$2 973 947	60
ECMO or tracheostomy, mv > 96 h, diagnoses except face, mouth and neck with major operating room (OR) procedures	$3 393 794	$3 393 794	100	$2 159 914	64	$1 341 138	$1 079 627	81	$1 034 341	77
Liver transplant, mcc	$2 829 588	$2 829 588	100	$2 829 588	100	$4 700 795	$4 700 795	100	$4 700 795	100
Cirrhosis and alcoholic hepatitis, mcc	$2 756 342	$2 699 797	98	$2 254 268	82	$1 981 554	$1 981 554	100	$1 425 571	72
Gastrointestinal hemorrhage, mcc	$2 685 614	$2 685 614	100	$2 590 319	96	$2 552 103	$2 552 103	100	$2 418 475	95
Gastrointestinal hemorrhage, cc	$2 555 010	$2 555 010	100	$2 409 815	94	$2 637 375	$2 637 375	100	$2 504 819	95
Heart failure and shock, mcc	$2 258 740	$2 112 563	94	$1 808 000	80	$2 487 166	$2 276 013	92	$1 523 836	61
Percutaneous and other intracardiac procedures without mcc	$1 909 875	$1 636 705	86	$1 162 242	61	$1 085 212	$846 238	78	$846 238	78
Red blood cell disorders	$1 833 081	$1 833 081	100	$1 477 525	81	$1 749 307	$1 749 307	100	$1 638 052	94
Red blood cell disorders, mcc	$1 767 578	$1 767 578	100	$1 702 515	96	$1 552 658	$1 552 658	100	$1 500 863	97
Peripheral vascular disorders, mcc	$1 717 868	$1 622 397	94	$1 461 745	85	$1 453 959	$1 393 628	96	$1 184 192	81
Peripheral vascular disorders, cc	$1 526 982	$1 485 954	97	$1 375 194	90	$1 283 404	$1 171 358	91	$1 046 972	82
Infectious disease, OR procedures, mcc	$1 444 822	$1 344 905	93	$1 052 519	73	$1 214 277	$1 047 848	86	$935 193	77
Disorders of liver (no malignancy, cirrhosis, or alcoholic liver disease), mcc	$1 409 463	$1 409 463	100	$1 210 205	86	$1 210 201	$1 210 201	100	$1 063 008	88
Other major cardiovascular procedures, mcc	$1 328 324	$1 055 666	79	$865 479	65	$1 086 436	$891 930	82	$603 268	56
Cirrhosis and alcoholic hepatitis, cc	$1 268 868	$1 268 868	100	$1 091 292	86	$1 044 998	$1 032 849	99	$759 125	73
Extensive OR procedures, mcc	$1 224 928	$1 112 679	91	$941 478	77	$2 293 443	$1 786 349	78	$1 532 752	67
Craniotomy and endovascular intracranial procedures, mcc	$1 517 652	$971 464	64	$971 464	64	$740 325	$385 395	52	$385 395	52
Psychoses	$1 168 476	$658 254	56	$658 254	56	$611 485	$322 580	53	$322 580	53
Intracranial vascular procedures, hemorrhage w/mcc	$1 130 902	$390 056	34	$390 056	34	$788 663	$788 663	100	$788 663	100
Percutaneous and other intracardiac procedures without mcc	$1 023 623	$857 399	84	$857 399	84	$1 285 074	$1 162 098	90	$639 871	50
Other circulatory system diagnoses, mcc	$906 382	$783 144	86	$783 144	86	$1 189 911	$1 160 926	98	$923 381	78

*Note*: Hematologic support is defined as intravenous iron infusion and/or red blood cell transfusion.

Patients with anemia accounted for a disproportionate percentage of leading inpatient costs, whether categorized by admission diagnosis or DRG. While representing 57% of HHT patients in 2022 and 56% in 2023, those with a diagnosis of anemia accounted for > 90% of the costs for 12 of the leading 16 inpatient cost drivers by admission diagnosis, and 15 of the 22 inpatient cost drivers categorized by DRG across years. Patients receiving hematologic support accounted for an even greater percent of cost relative to their population size. While representing only 25% and 24% of HHT patients in 2022 and 2023, patients receiving hematologic support accounted for > 75% of the costs for nine and 10 of the 16 leading inpatient cost drivers by admission diagnosis in 2022 and 2023 respectively, and 13 of the 22 leading inpatient cost drivers by DRG across years.

The percentage of patients with liver transplants in this population is noteworthy. Among patients with HHT, the prevalence was 0.3% in 2022 and 0.4% in 2023. Among patients receiving hematologic support, the prevalence was 1.2% and 1.4% respectively. This is 40 times higher than the prevalence of liver transplant in the US population (0.03%) [19].

#### 3.3.3 Pharmacy Costs

The leading pharmaceuticals were grouped by indication for ease of interpretation (Table 6). Of the eight indication areas, four are related to HHT and HHT comorbidities: HHT/antiangiogenic, pulmonary arterial hypertension, anticoagulation, and hepatic encephalopathy. Four areas are not specific to HHT: solid tumor oncology; angioedema; Type 2 diabetes mellitus; and autoimmune diseases including Crohn's, ulcerative colitis, and rheumatoid arthritis. Pharmacy costs attributable to the four indication areas related to HHT approximated $13 million in 2022 and 2023.

**TABLE 6:** Direct pharmacy costs by major indication in 2022 and 2023 in patients with HHT and subgroups with anemia and IV iron and/or red blood cell transfusion. Highlighted major indications are specifically related to HHT and HHT comorbidities. [Color table can be viewed at wileyonlinelibrary.com]

Generic names	Indications	2022					2023				
		All HHT	Anemia		Hematologic support		All HHT	Anemia		Hematologic support	
		*n* = 24 407 (100%)	*n* = 13 856 (57%)	%	*n* = 6102 (25%)	%	*n* = 23 524 (100%)	*N* = 13 192 (56%)	%	*n* = 5726 (24%)	%
C1 esterase inh, lanadelumab‐flyo, icatibant, berotralstat	*Angioedema*	$4 882 146	$3 630 300	74	$3 630 300	74	$2 957 685	$1 942 640	66	$1 942 640	66
Selexipag, macitentan	*Pulmonary arterial hypertension*	$4 643 407	$4 027 507	87	$2 329 870	50	$4 733 739	$3 797 479	80	$3 082 891	65
Trastuzumab	*Solid tumor/oncology*	$4 284 905	$4 284 905	100	$4 284 905	100	$4 085 431	$4 085 431	100	$4 085 431	100
Pomalidomide, pazopanib, lenalidomide	*HHT/angiogenic*	$3 588 424	$3 337 043	93	$2 957 833	82	$3 050 006	$2 773 786	91	$2 467 236	81
Semaglutide, tirzepatide, empagliflozin, Dulaglutide	*Type 2 diabetes mellitus*	$3 543 052	$2 471 802	70	$1 170 209	33	$6 337 175	$4 452 675	70	$1 832 488	29
Adalimumab, ustekinumab	*Crohn's/ulcerative colitis/arthritis*	$3 076 492	$2 418 771	79	$941 404	31	$2 661 716	$2 277 070	86	$1 076 616	40
Apixaban, rivaroxaban	*Anticoagulation*	$2 995 217	$2 108 252	70	$1 212 982	40	$3 308 240	$2 280 343	69	$1 215 530	37
Rifaximin	*Hepatic encephalopathy*	$1 878 186	$1 811 878	96	$1 246 906	66	$1 891 624	$1 865 892	99	$1 307 947	69

*Note*: Hematologic support is defined as intravenous iron infusion and/or red blood cell transfusion.

In 2022, patients with anemia were over‐represented in all drug categories, accounting for 70%–97% of costs. In 2023, patients with anemia accounted for ≥ 70% of cost in six of the eight categories, though still accounting for 66%–69% of the costs in angioedema and anticoagulation. Patients with hematologic support accounted for ≥ 70% of costs in three categories in 2022: HHT/antiangiogenic, angioedema, and solid tumor/oncology; and two in 2023: HHT/antiangiogenic and solid tumor/oncology. Patients receiving hematologic support accounted for < 40% of drug costs in three categories: type 2 diabetes mellitus, autoimmune disease (Crohn's/ulcerative colitis/arthritis), and anticoagulation.

#### 3.3.4 Comparison to Other Rare Diseases

**FIGURE 2:**
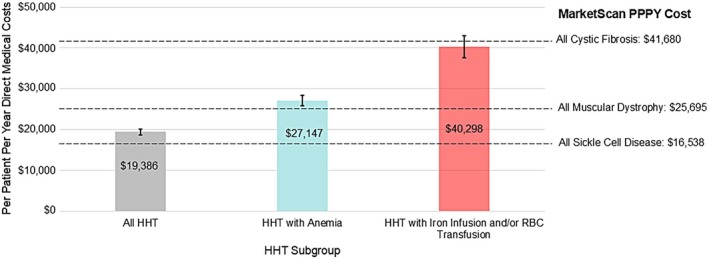
Mean (SE) per patient per year direct medical costs (all healthcare costs) in 2022 in patients with HHT and subgroups with anemia and hematologic support versus comparable rare diseases [17]. [Color figure can be viewed at wileyonlinelibrary.com]

Figure 2 shows the mean ± SE PPPY costs (all healthcare costs) for patients with HHT, HHT with anemia, and HHT receiving hematologic support from this study compared to a recent analysis of rare disease costs in the MarketScan Research claims database [17]. MarketScan costs were inflated from 2020 (the last year in the analysis) to 2022 dollars using a medical inflation calculator.

## 4 Discussion

The direct medical costs of treating patients with HHT have not been well‐described, with very limited data published prior to this study [16, 17]. This is the first study to provide a detailed description of healthcare resource utilization and cost, replicated over time. In 2022–2023, the average PPPY healthcare cost of patients with HHT was > $19 000 across years.

As demonstrated in the present study, the primary cost driver in patients with HHT is bleeding and its complications, namely anemia. The mean PPPY total direct medical cost for all patients with HHT ($19 386) was approximately 20% greater than those reported for patients with sickle cell disease ($16 538) [17]; the PPPY cost of treating HHT patients with anemia ($27 147) was comparable to that reported for treating patients with muscular dystrophy ($25 147); and the PPPY cost of treating patients with HHT receiving hematologic support with intravenous iron and/or red cell transfusion ($40 298) was comparable to that for treating patients with cystic fibrosis ($41 680) [17]. Anemia is a serious and often underappreciated consequence of HHT, constantly recurring in these patients due to chronic bleeding complications, and may become severe enough to result in end‐organ damage or acute coronary syndromes. While recurrent epistaxis clearly contributes to anemia, patients with the most severe anemia in HHT typically have chronic gastrointestinal bleeding. Additionally, given that a recent study of over 600 women with HHT found rates of heavy menstrual bleeding exceeding 70%, the possible contribution of heavy menstrual bleeding to anemia in HHT must be considered [1].

In addition to hematologic support with intravenous iron and red cell transfusion, we found significant use of systemic bevacizumab, pazopanib, and immunomodulatory imide drugs, with considerable associated pharmacy and outpatient costs. Moreover, procedures to control bleeding were also among the top drivers of outpatient cost. The use of expensive antiangiogenic drugs is only increasing as more evidence emerges regarding their efficacy in HHT, resulting in slowly expanding off‐label access [20, 21]. But while the use of expensive drugs may be expected to increase overall cost in HHT, it should be noted that systemic bevacizumab to treat bleeding in HHT was found to be cost saving in a rigorous cost‐effectiveness analysis, highlighting the even greater costs of hematologic support, emergency care, and hospital admissions for bleeding and anemia [22]. In a disease where adequate systemic therapy may convert an iron infusion and/or red‐cell transfusion dependent patient into one liberated from requiring hematologic support over the course of a few months, the fact that such a high proportion of the patients in this sample required hematologic support highlights the limitations of the current off‐label drugs and confirms the need to develop disease‐modifying therapeutics, especially those aimed to address the root cause of HHT.

That anemia and its severity is associated with incremental cost has been well‐documented in multiple disease states, such as chronic kidney disease (CKD) and heart failure [23, 24, 25, 26]. In a sample of 54 701 patients with CKD in the Henry Ford Health System, 23% had anemia versus 56%–57% of HHT patients in this study [23]. Among those patients with anemia, 31% of the CKD patients received either hematologic support or an erythropoiesis stimulating agent, versus the 43%–44% of HHT patients with anemia receiving hematologic support in this study. The 1‐year risk of hospitalization for heart failure in the patients with CKD and anemia was more than three times that of patients without anemia (HR = 3.45), while in this study, 95%–96% of the costs of hospitalization for heart failure (defined by DRG) were in patients with anemia. In HHT, it is well recognized that the high‐output heart failure relatively common in patients with liver AVMs is clearly exacerbated by anemia and improved by its resolution [27, 28]. Further, in an analysis of 842 patients with HHT identified from the National Inpatient Sample database, 15.7% of patients had a comorbid diagnosis of congestive heart failure, which was statistically significantly associated with HHT [29]. In a study of 596 456 patients admitted to the hospital for heart failure in California, 27.1% of patients had anemia and 6.2% received transfusion; those with anemia severe enough to require transfusion had a significantly increased risk of death (OR = 3.81) [30]. In the studies cited, anemia was shown to more than double the risk for cardiovascular events, a finding reported in multiple systematic reviews [31, 32]. The fact that the majority of treatment for cardiovascular comorbidities in this study occurred in the HHT patients with anemia and/or receiving hematologic support suggests that anemia may be a primary contributing factor to cardiovascular complications in HHT as well.

Unfortunately, undertreatment of anemia with intravenous iron remains common in HHT, raising further concerns regarding the potential for its contribution to morbidity in HHT [13, 33]. In the present study, while only 50% of patients with HHT and anemia received hematologic support, it is highly likely that a significant portion of the anemic patients were not adequately treated, leaving patients at risk for complications. Moreover, given systemic undertreatment of anemia in HHT, the costs related to anemia in this study are very likely to be underestimated.

In addition to bleeding and anemia, we found that liver AVMs also result in a high cost burden in patients with HHT. While a minority of patients with HHT ultimately develop complications from liver AVMs, these complications are usually serious or fatal [5]. In a study of 1286 patients with HHT recruited at 14 HHT centers of the Brain Vascular Malformation Consortium, a history of anemia (HR = 2.93), gastrointestinal bleeding (HR = 2.63), and symptomatic liver AVMs (HR = 2.10) were associated with an increased risk for mortality, independent of age. As our sample identified a large subset of patients with chronic anemia, a significant portion of HHT patients remain at risk for highly morbid or mortal events, further highlighting that current treatments do not address the most at‐risk complications associated with HHT [34]. Moreover, while liver AVMs are categorized as a nonbleeding complication of HHT, hemostatic complications of the chronic liver disease that they may cause, including coagulopathy and thrombocytopenia, may worsen bleeding manifestations. In the present study, cirrhosis and complications of portal hypertension, including hepatic encephalopathy, were also demonstrated as leading cost drivers among HHT patients with anemia and those receiving hematologic support. Indeed, 80%–87% of the cost of drugs used to treat portal hypertension were in patients with anemia. The high prevalence of liver transplantation in this study is also noteworthy. Among patients with HHT receiving hematologic support, the prevalence of liver transplantation was 40 times higher than among the general US population [19]. The costs attributed to alcohol‐associated liver disease in these patients is also of interest, as there is anecdotal evidence from US HHT Centers of Excellence that liver disease in these patients may be misdiagnosed as alcohol‐associated given the requirement for specific advanced imaging studies necessary to diagnose liver involvement with diffuse AVMs. Within these centers, it has been reported that 67%–74% of HHT patients have liver AVMs when screening with multislide CT scans (M.C., personal communication). The distribution of patients receiving care at healthcare centers without HHT expertise may contribute to the high rate of cirrhosis diagnosis.

Our study has important limitations, primarily related to the limitations associated with using claims data. These data represent patients with US health insurance, which, while representing 92% of the US population, does not represent the entire population [35]. The database does not include or represent patients treated with insurance such as the Department of Defense, Veterans Affairs, or the Indian Health Service, and it does not represent the treatment of patients outside of the United States. The database does not include detailed provider notes, and therefore this granular data was not available to us. The absence of detailed clinical outcome data also limits the ability to correlate cost with treatment efficacy, years of productivity, and certain markers of disease severity. Despite these claims data‐associated limitations, the database is highly representative of direct medical costs paid by commercial insurers, Medicare, and Medicaid in the United States. The specific approach used in this study to identify the patient population utilizing the I78.0 ICD‐10 code for HHT has not been specifically validated, as there is a dearth of research in this space, and to perform this study we had to develop a rigorous population sample selection process. Our sample selection process prioritized specificity, such that the population of patients identified as HHT was likely to have HHT and not likely to have simply been evaluated to rule out HHT. Perhaps the most important limitation was that the patients in this sample are only those diagnosed with HHT by a healthcare provider (and characterized by the HHT ICD 10 code) and do not include undiagnosed patients. It is well recognized that HHT is an underdiagnosed and neglected inherited bleeding disorder [36, 37], and patients without a diagnosis cannot be analyzed. While a prior study concluded that approximately 90% of patients with HHT were undiagnosed, progress in the intervening time, including the establishment of numerous HHT Centers of Excellence and increased awareness among hematologists and other providers, has likely improved on this woeful number. However, many patients remain undiagnosed well into adulthood, often despite clear bleeding manifestations and AVM complications, and the delay in HHT diagnosis from the initial presentation of disease‐specific signs and symptoms remains unacceptably long, at nearly three decades [38].

In conclusion, the PPPY cost of care in patients with HHT is substantial, eclipsing those of patients with other resource intensive diseases such as sickle cell disease. These costs are driven primarily by bleeding, its consequences, and the medical and procedural hemostatic therapies to treat them. Patients with HHT and anemia, and especially those with more severe anemia receiving hematologic support with iron infusion and RBC transfusion, are at significant risk for comorbid events with meaningful incremental healthcare resource use and higher direct medical costs. There is a tremendous need for new treatments to reduce the complications associated with HHT and improve quality of life [16, 17].

## Author Contributions

Eric Duhaime, Hanny Al‐Samkari, and Tracy J. Mayne contributed to study design. Hanny Al‐Samkari, Eric Duhaime, Hemant Patle, Misty Troutt, and Tracy J. Mayne contributed to analyses. Hanny Al‐Samkari, Eric Duhaime, Marianne Clancy, Tracy J. Mayne, Misty Troutt, and Hemant Patle contributed to the interpretation of results. Tracy J. Mayne and Hanny Al‐Samkari were the primary manuscript writers with contributions from Eric Duhaime, Marianne Clancy, Misty Troutt, and Hemant Patle.

## Ethics Statement

This study was IRB exempt as it involved database analysis of fully anonymized data.

## Conflicts of Interest

Eric Duhaime is an employee of Diagonal Therapeutics, which funded this study. Tracy J. Mayne, Misty Troutt, and Hemant Patle were paid consultants on this study but do not own stock or other financial interests in Diagonal Therapeutics. Hanny Al‐Samkari receives research funding from Agios, Amgen, Novartis, Sobi, Vaderis; and is a consultant for Agios, Alnylam, Amgen, Alpine, argenx, Diagonal, Novartis, Pharmacosmos, Sanofi, Sobi. Marianne Clancy receives educational funding from Diagonal Therapeutics and is an Advisory Board member for Alnylam.

## Supporting information


**Data S1.** Supporting Information.

## Data Availability

For data requests, please email hal-samkari@mgh.harvard.edu.
