# Supplementary material for: Characterizing the Healthcare Utilization and Costs of Hereditary Hemorrhagic Telangiectasia
Source: Am J Hematol. 2025 Jul 2;100(10):1722–35. doi: 10.1002/ajh.27756 (PMC12417757; doi:10.1002/ajh.27756)
Supplement: Supplementary file 1 — Data S1. Supporting Information. [file AJH-100-1722-s001.docx]

**Supplemental Table 1. ICD 10, ICD 10 PCS, CPT and HCPCS codes defining anemia, iron infusion and iron transfusion.**

**Anemia**

ICD 10 Codes: D50, D62, D63, D649, D52, D51, D6489, D464, D53, D508, D509, D529, and D539

**Transfusion**

CPT Codes: 36430, 36440, 36560, 36455, and 36456

HCPCS Codes: P9010, P9011, P9016, P9017, P9019, P9020, P9021, P9022, P0923, P9031, P9032, P9033, P9034, P9035, P9036, P9037, P9038, P9039, P9040, P9043, P9044, P9048, P9050, P9051, P9052, P9053, P9054, P9055, P9056, P9057, P9058, P9059, P9060, P9070, P9071, P9073, and P9099

PCS Codes: 30233H, 30233H, 30233K, 30233L, 30233M, 30233N, 30233P, 30233R, and3027

Revenue Code: 0391

**Iron infusion**

HCPCS Codes: J1437, J1439, J1750, J2916, Q0138, and Q0139
